# Analysis of the Long-term Visual Outcomes of ForeseeHome Remote Telemonitoring: The ALOFT Study

**DOI:** 10.1016/j.oret.2022.04.016

**Published:** 2022-04-26

**Authors:** Mariam Mathai, Shivani Reddy, Michael J. Elman, Richard A. Garfinkel, Byron Ladd, Alan L. Wagner, George E. Sanborn, Jennifer H. Jacobs, Miguel A. Busquets, Emily Y. Chew

**Affiliations:** 1 Retina Group of Washington, Greenbelt, Maryland.; 2 Retina Associates of Kentucky, Lexington, Kentucky.; 3 Elman Retina Group, Glen Burnie, Maryland.; 4 Virginia Eye Institute, Richmond, Virginia.; 5 Wagner Macula & Retina Center, Virginia Beach, Virginia.; 6 Notal Vision Monitoring Center, Manassas, Virginia.; 7 Division of Epidemiology and Clinical Applications, National Eye Institute, National Institutes of Health, Bethesda, Maryland.

**Keywords:** Anti-VEGF, Artificial intelligence, Home monitoring, Macular degeneration, Retina, Telemedicine

## Abstract

**Purpose:**

To evaluate long-term visual acuity (VA) and performance of a monitoring strategy with a self-operated artificial-intelligence–enabled home monitoring system in conjunction with standard care for early detection of neovascular age-related macular degeneration (nAMD).

**Design:**

Retrospective review.

**Subjects:**

Patients with dry-age-related macular degeneration from 5 referral clinics.

**Methods:**

Clinical data of patients monitored with ForeseeHome (FSH) device from August 2010 to July 2020 were reviewed.

**Main Outcome Measures:**

Visual acuity at baseline, VA at diagnosis of nAMD for eyes that converted while monitored, and VA from the final study follow-up, weekly frequency of use, duration of monitoring, modality of conversion diagnosis (system alert vs. detection by other standard care means), and duration and number of treatments since conversion to final study follow-up were collected.

**Results:**

We reviewed 3334 eyes of 2123 patients with a mean (standard deviation [SD]) age of 74(8) years, monitored for a mean (SD) duration of 3.1 (2.4) years, with a total of 1 706 433 tests in 10 474 eye-monitoring years. The mean (SD) weekly use per patient was 5.2 (3.4), and it was persistent over the usage period. Two hundred eighty-five eyes converted while monitored at an annual rate of 2.72% and were treated with a mean (SD) 17.3 (16.5) injections over a mean (SD) 2.7 (2.0) years, with 6.4 (3.1) injections per year for eyes treated for > 1 year. The median VAs at baseline and at final follow-up for eyes that did not convert were 20/27 and 20/34 with a median change of 0.0 letters. The median VAs at baseline, conversion, and final follow-up for eyes that converted during the monitoring period were 20/30, 20/39, and 20/32 with a median change from baseline to conversion, baseline to recent, and conversion to recent of −4, −4, and 0 letters, respectively. Fifty-two percent of conversions detected had a system alert before conversion. Forty-eight percent of patients were detected by symptoms or routine visit. Patients experienced a non-nAMD alert on average every 4.6 years. At conversion and at final follow-up, the proportion (95% CI) of eyes that maintained ≥20/40 was 84% (78% to 88%) and 82% (76% to 86%), respectively.

**Conclusions:**

Patients in the FSH monitoring program showed excellent long-term VA years after conversion to nAMD.

Age-related macular degeneration (AMD) is the leading cause of vision loss among patients 50 years of age and older and affects > 8 million people in the United States.^1–3^ Although neovascular AMD (nAMD) represents only 10% to 15% of all AMD, it is responsible for > 80% of all AMD-related vision loss.^4,5^ Early detection of conversion from intermediate AMD (iAMD) to nAMD is essential for preservation of visual acuity (VA), as VA at the time of diagnosis of nAMD is a strong predictor of long-term visual outcomes.^6–8^

To promote early detection of conversions, home monitoring is often used between routine office visits. Home monitoring may include the use of the Amsler grid, as well as various smartphone applications,^9,10^ all of which detect changes in metamorphopsia attributable to changes in macular architecture. Home OCT devices are currently under development that may play a future role in the early detection and monitoring of nAMD.^11,12^

The ForeseeHome (FSH) monitoring program is a home monitoring strategy that incorporates symptom realization leading to requests for a prompt visit and prescheduled office visits as part of a standard care. The FSH device (ForeseeHome, Notal Vision Inc), cleared by the Food and Drug Administration in 2009, is an artificial-intelligence–enabled visual field analyzer based on hyperacuity (or vernier acuity), a characteristic of the human visual system involving perception of even the most minute differences in the relative localization of 2 objects in space.^13^ Hyperacuity is very sensitive to the misalignment of photoreceptors and is therefore useful in objective quantification of perceived visual distortions, that is, metamorphopsia.

The FSH is prescribed to patients diagnosed with iAMD and VA of 20/60 or better in the monitored eye. The Notal Vision Monitoring Center (NVMC) is an independent diagnostic testing facility accredited by Medicare that functions as a digital healthcare provider. It is responsible for verifying insurance benefits, FSH device provisioning, and clinical monitoring of FSH data of patients who are referred. The NVMC provides training for patients on the use of the device, troubleshoots device issues, provides adherence reminders, monitors clinical outcomes to assess device efficacy, and communicates with the patients’ eye-care providers to report FSH alerts. A baseline is required to be established for each eye before monitoring is initiated.

The Home Monitoring of the Eye (HOME) study, conducted in association with the Age-Related Eye Diseases Study 2 (AREDS2), was a robust prospective, multicenter, masked, and randomized trial documenting the efficacy of FSH in identifying eyes converting to nAMD in a clinical study setting.^14–17^ This trial showed that, among eyes that converted from iAMD to nAMD, those assigned to a strategy that included daily monitoring with the FSH in conjunction with standard care (symptoms realization and routine office visits) were diagnosed sooner and lost less vision (median change from baseline of −4 ETDRS letters) than eyes assigned to standard care monitoring alone (−9 ETDRS letters; *P* = 0.021).

Efficacy observed in prospective clinical trials does not always translate to real-world effectiveness for numerous reasons,^18^ and evaluation of real-life data in combination with trial data is often needed to appropriately evaluate the efficacy of any medical intervention. Analysis of data collected from FSH monitoring in a real-world setting was reported by Ho et al.^19^ This review validated that eyes that were detected early with the home monitoring strategy maintained good vision, as reflected primarily in a median loss of 3 letters from baseline to conversion of 306 eyes and in a variety of other indices. The study concluded that the structured implementation of the FSH system, including telemonitoring supervision by the NVMC, resulted in real-world performance similar to those reported from the HOME randomized clinical trial. However, both the HOME study and the real-world performance study were limited to time of diagnosis with nAMD, and the follow-up period did not include data collection from later time points following years of treatment.

Several studies have reported long-term outcomes of eyes undergoing treatment of nAMD. The Seven Up study^20^ reported that, at a mean of 7.3 years (range, 6.3–8.5 years) after entry into Anti-VEGF Antibody for the Treatment of Predominantly Classic Choroidal Neovascularization in AMD (ANCHOR) or Minimally Classic/Occult Trial of the Anti-VEGF Antibody Ranibizumab in the Treatment of Neovascular AMD (MARINA) studies, 37% of study eyes met the primary end point of 20/70 or better, best-corrected VA, with only 23% achieving a best-corrected VA of 20/40 or better. The Comparison of AMD Treatments Trials 5-year outcomes^21^ showed that vision gains that were made during the first 2 years of treatment were not maintained at 5 years follow-up; however, 50% of eyes had VA of 20/40 or better, solidifying anti-VEGF therapy as a major long-term therapeutic advance for nAMD.

Real-world findings from the Long-term Follow-up of Patients with Exudative AMD Treated with Intravitreal Anti-VEGF Injections study^22^ further emphasized the link between consistent treatment and maintenance of long-term vision. A study of Long-term Outcomes of Treat and Extend Regimen of Anti-VEGF in nAMD^23^ concluded that most patients (74%) improve or maintain VA long term using a treat and extend model, with only 45.1% achieving 20/50 or better VA with sustained treatment. Mean baseline VA was 20/100, suggesting that maintenance of vision does not translate to good, functional vision.

These findings raise the possibility that, for some nAMD patients, there is a negative feedback loop starting with suboptimal initial vision at conversion, leading to nonpersistence and decreased adherence to the treatment schedule,^24,25^ which further leads to a decreased vision and reduction in the motivation to maintain proper follow-up.

This study provides confirmatory evidence of the usage patterns and VA at the time of conversion to nAMD while monitored with the FSH system and reports long-term VA data after years of treatments with anti-VEGF therapy in a real-world setting, proposing an alternative long-term path for patients who adopted this digital healthcare service.

The study was initiated by retina physicians in 5 high-prescribing retina clinics. Over years of following patients who used the FSH system, these physicians developed a hypothesis that the eyes that converted while monitored at home with the system consistently maintained good functional and anatomic status with persistent management. The Analysis of the Long-term Visual Outcomes of FSH Remote Telemonitoring (ALOFT) study was designed to examine that hypothesis.

## Methods

This retrospective review included the medical records of all patients who established valid baseline test results and who were monitored with FSH from 5 high-referring clinics from August 2010 to July 2020. An institutional review board (IntegReview) review in September 2020 determined that, because this was a retrospective analysis of existing, deidentified data, an exemption was granted on the basis that the study satisfied exemption category 4 (ii).

Two data sources were predefined and integrated during the analysis. Data were collected from medical records at the clinics and included demographics, dates, and VAs at baseline, at diagnosis of nAMD for eyes that converted while monitored, and from the visit closest to the chart review (termed as final follow-up), number of treatments since conversion to final follow-up, and the AMD status of the fellow eye. The VA data reported during the data collection were measured with a Snellen chart during the office visits, were converted to logarithm of the minimum angle of resolution for the purpose of the analysis, and are presented in logarithm of the minimum angle of resolution, ETDRS letters, and Snellen equivalents, where applicable.

Data collected from the NVMC included the proportion of eyes that established a baseline, the count and timing of all tests performed with the device, and the duration of monitoring from the first to last test. For eyes that converted, the modality that triggered the diagnosis of nAMD was collected, that is, system alert versus detection by other standard care means (which may include the realization symptoms by the patient leading to a call and request for a visit or to a routine prescheduled office visit) and the number of non-nAMD system alerts.

Statistical analyses included descriptive statistics with comparison between subcohorts and longitudinal analyses, including correlation over time and a survival analysis. Several outcome measures were calculated: the duration of monitoring, the weekly frequency of use of the device, the number and rate of conversions, the duration of follow-up from conversion to final study follow-up, and the number of treatments in that period.

The primary outcome measure was the change in VA from the office visit upon enrollment to the FSH program until the recent office visit for eyes that converted to nAMD while monitored with FSH. Also evaluated was the change in VA from baseline to time of conversion, from time of conversion to the final follow-up, and from baseline to final follow-up for eyes that did not convert while monitored. Secondary analyses included the proportion of eyes that maintained functional vision of 20/40 or better at conversion and at the final follow-up and the mean annual number of injections from conversion to final follow-up. The proportion of conversions identified in a visit triggered by a device alert and in relation to the nAMD status of the fellow eye and the annual rate of non-nAMD alerts were analyzed.

With the objective of maximizing the reported follow-up period, information was collected for a variable duration for each participant, and the analysis methods were adopted accordingly. Several outcomes were evaluated longitudinally. The persistence of the weekly frequency of use of the device over the duration of monitoring was determined. The predicted duration of participation in the monitoring program from the first test to the last test with the device (regardless of the AMD status at that stage) was calculated using a Kaplan–Meier survival analysis. The time gaps between the data lock to the last reported visit to the retina clinic by participants who were diagnosed with nAMD were calculated to evaluate the persistence of follow-up during the treatment phase.

The data collected in the study included the overall number of treatments from conversion to final follow-up and the resulting change in VA. The distribution of these periods, ranging from a few weeks to 10 years, allowed for the evaluation of the long-term consistency and efficacy of the management of these eyes.

## Results

**Figure 1.**
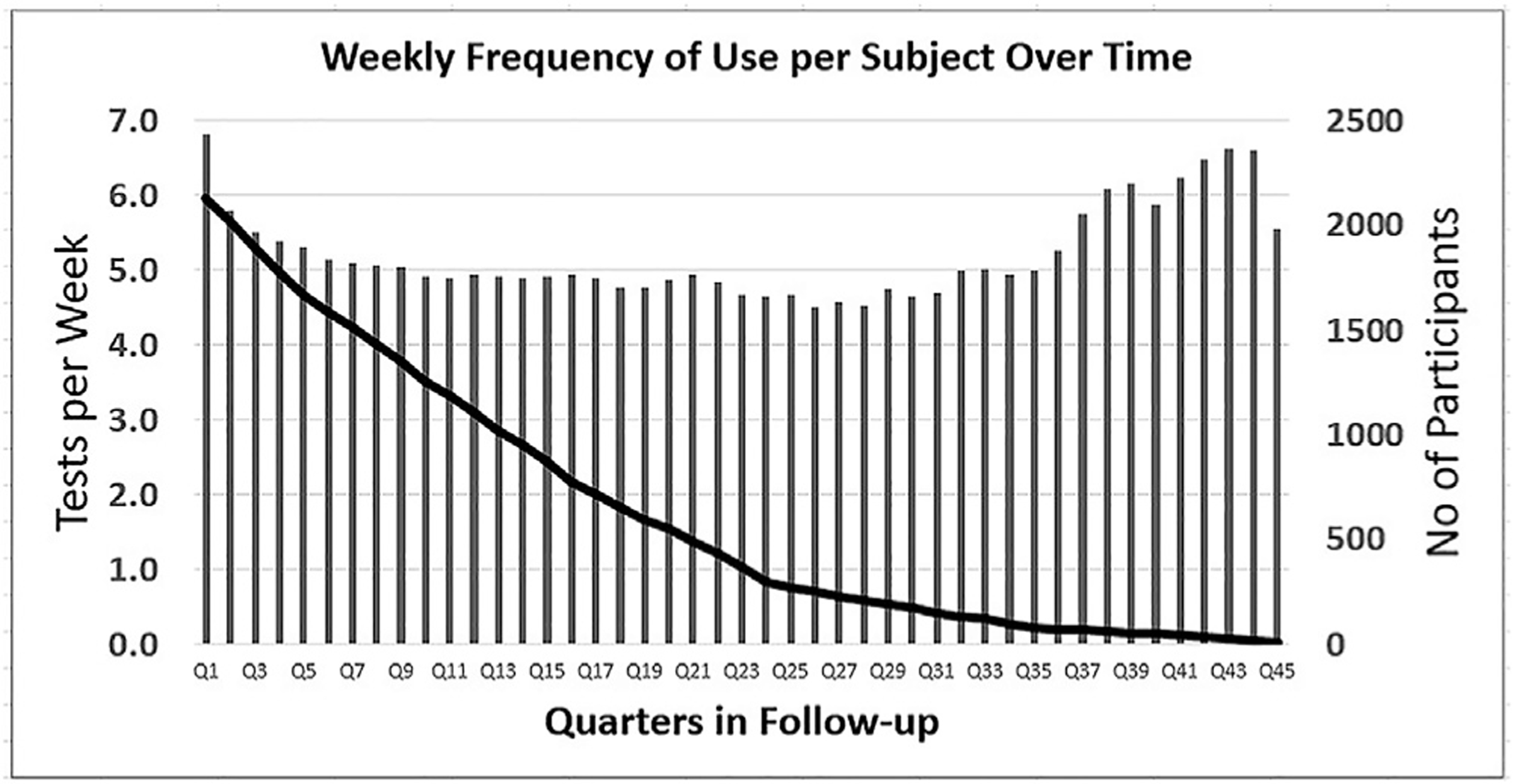
Number of users and average weekly frequency of use per quarter of participation in the program.

During the baseline process before entry into the study, 82.9% (81.6%–84.1%; 95% confidence interval [CI]) of eyes established a baseline. Data from 3334 eyes of 2123 participants from 5 retina clinics in the US that established a baseline were collected (Table 1). Twelve hundred and eleven (57%) participants enrolled with both eyes, and 912 (43%) enrolled with a single eye. Monitoring data were collected from the participants during a period of mean (standard deviation [SD]) 3.1 (2.4) years, with the longest standing participant monitored for over 10 years. All the participants cumulatively performed a total of 1 706 433 tests in 10 474 eye-monitoring years. The mean (SD) weekly frequency of use of the device per patient was 5.2 (3.4). The mean weekly frequency of use of the device was persistent throughout the usage period (Fig 1) with the lowest quarter at a mean 4.7 tests per week. Two hundred and eighty-five eyes converted while monitored at an annual rate of 2.72% and were treated with a mean (SD) 17.3 (16.5) injections over mean (SD) 2.7 (2.0) years, yielding a mean (SD) of 6.4 (3.1) injections per year for eyes treated for > 1 year.

Evaluation of treatment persistence showed that, of the 285 converted eyes, 31 (11%) and 19 (7%) had their last office visit > 6 or 12 months before the data lock, respectively. For the primary outcome of the study, the logarithm of the minimum angle of resolution mean (SD) and median (interquartile range) and Snellen equivalent of the mean and median VA at baseline of the 285 eyes that converted to nAMD while monitored were 0.19 (0.16), 0.18 (0.1–0.3), 20/31, and 20/30 respectively, and the mean (SD) and median (interquartile range) change in VA from baseline to the final follow-up were −7.0 (18.0) and −4.0 (−11.0 to 3.0) ETDRS letters equivalent, respectively. Table 2 presents VA results at baseline, at conversion when applicable, and at final follow-up; the percentage maintaining functional vision of 20/40 or better; the change in VA; and the respective durations by group. Of note, the baseline VA of eyes that eventually converted was 2.5 letters worse than the eyes that did not convert while monitored (*P* = 0.014).

Of the 285 conversions to nAMD detected by the home monitoring strategy, 52% of the conversions were detected during a visit triggered by a system alert, whereas 48% were detected during a visit triggered by standard care means, including symptoms realization and detection at routine, prescheduled visits. A breakdown of this information was not collected during the routine care of the participants. There were no statistical or clinically significant differences in the VA outcomes between converted eyes detected by device alert or standard care means, with the same baseline VA of 20/30 and VA change from baseline to conversion, from baseline to final follow-up, and from conversion to final follow-up of −4, −4, and 0 letters versus −3.5, −4.5, and 0 letters (*P* = 0.66, 0.97, and 0.47), respectively. The mean (SD) number of injections for these groups was similar at 17.7 (17.8) versus 17.0 (15.0) (*P* = 0.76). The proportion of detections due to device alerts increased to 56% when the fellow eye was diagnosed with iAMD and decreased to 47% when the fellow eye was diagnosed with nAMD. This difference was not statistically significant (*P* = 0.12). There were no statistically or clinically significant differences in the VA outcomes related to the status of the fellow eye. Non-nAMD alerts were issued to eyes that did not convert to nAMD, which averaged (95% CI) every 4.6 (4.4–4.8) years.

**Figure 2.**
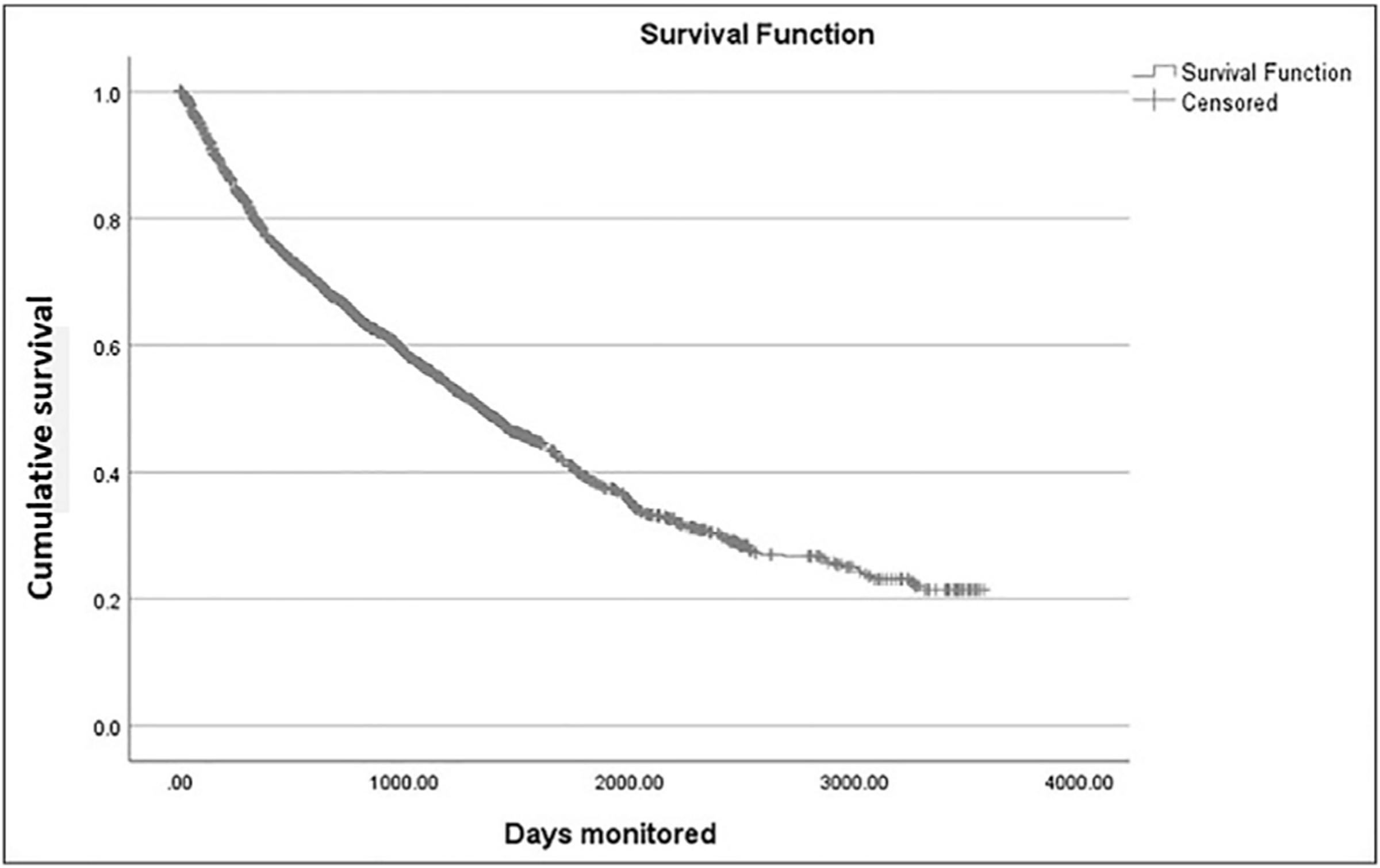
Kaplan–Meier survival function. Survival time represents the period of participation in the home monitoring program.

Kaplan–Meier survival analysis was performed to estimate the expected duration of participation in the home monitoring program. Of the 2123 participants, 1101 (52%) left the program before the data collection date. Reasons for leaving the program included conversion to nAMD, a non-nAMD alert, a consecutive failure to establish a new baseline, or an unspecified decision by the user. At the arbitrary time point of data lock, 1022 (48%) of the patients were still monitoring with the device; because of the lack of information about the upcoming period of usage with the device, they were labeled as censored. The predicted mean and median (95% CI) time of participation in the program (i.e., survival) were 4.5 (4.3–4.7) and 3.7 (3.4–4.0) years, respectively. The Kaplan–Meier survival function is presented in Figure 2.

**Figure 3.**
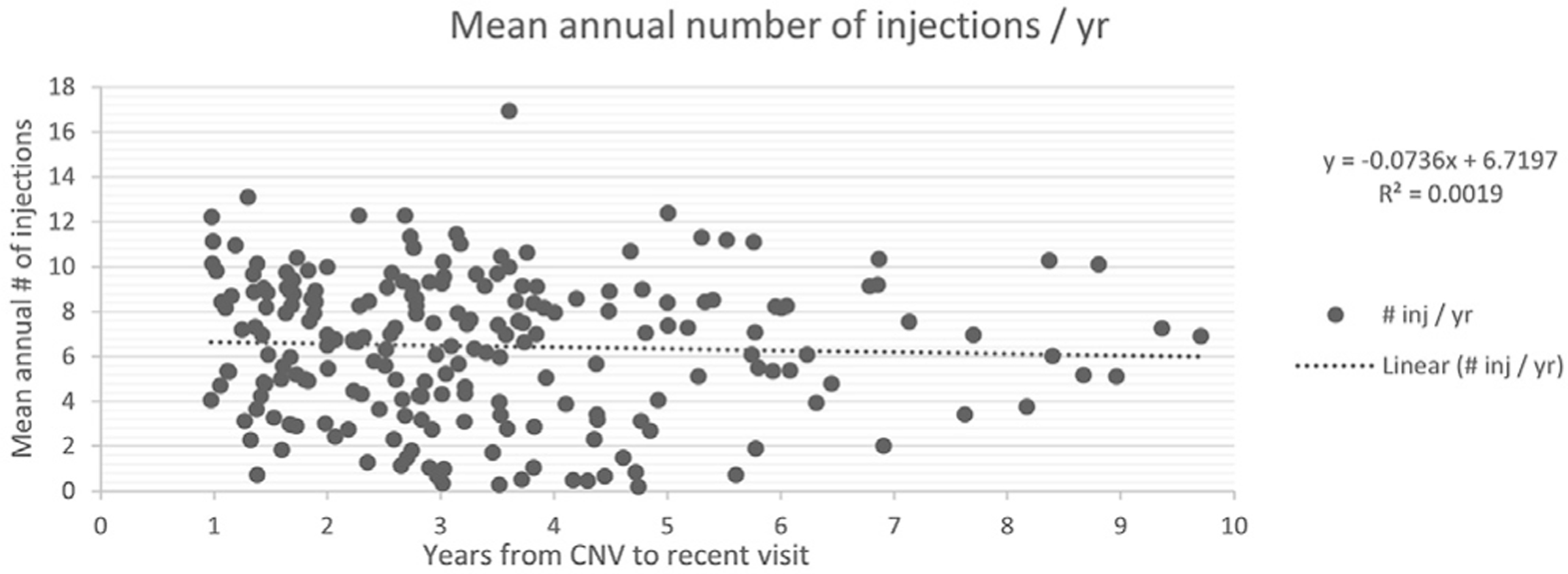
Mean annual number of anti-VEGF injections by time from conversion. CNV = choroidal neovascularization.

**Figure 4.**
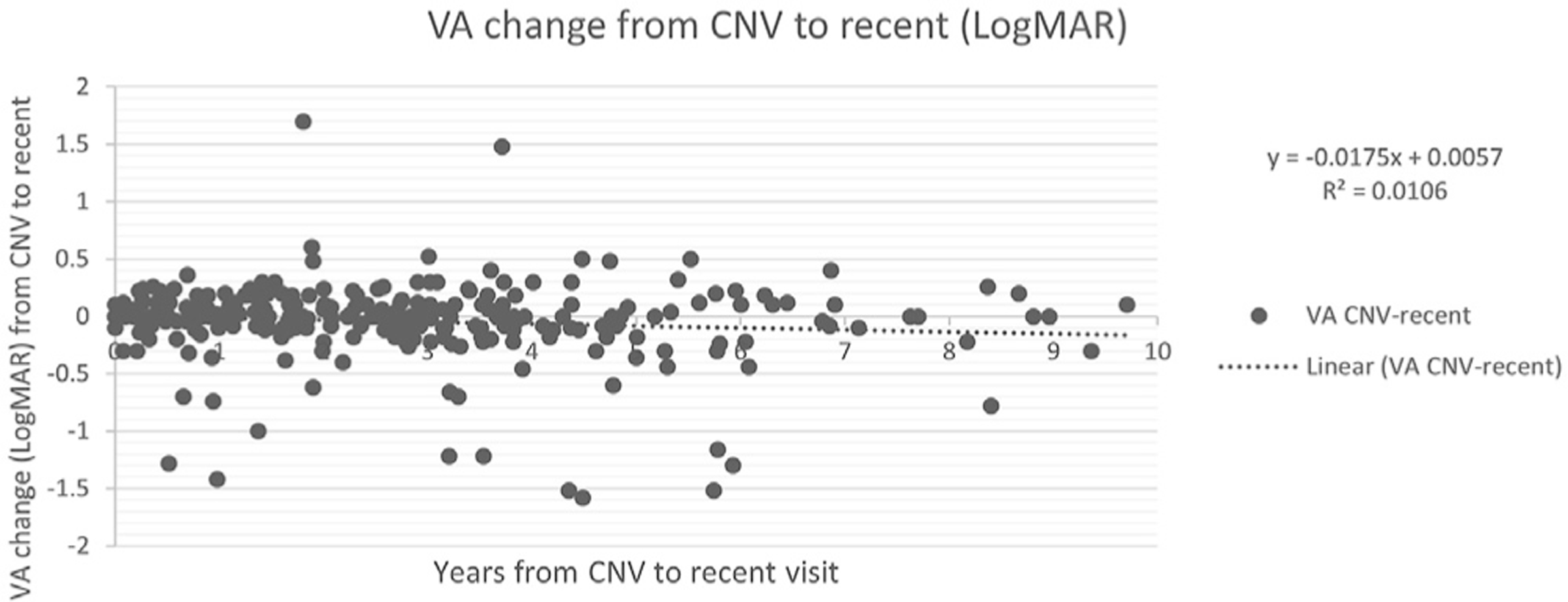
Visual acuity change from conversion to final study follow-up by time from conversion. CNV = choroidal neovascularization; VA = visual acuity.

We evaluated the long-term consistency and efficacy of treatments of all the eyes diagnosed with nAMD while using the home monitoring strategy. No correlation was found between the mean annual number of injections for eyes with > 1 year since the conversion to nAMD versus the time from the conversion to final follow-up (*R^2^* = 0.0019, *P* = 0.53) (Fig 3). No correlation was found between the change in VA versus the time from the conversion to nAMD to final follow-up (*R^2^* = 0.0106, *P* = 0.09) (Fig 4).

## Discussion

The ALOFT study results provide for the first time evidence of the long-term vision outcomes of iAMD patients who received an FSH prescription from their retina specialist, enrolled in the home monitoring program, converted to nAMD while participating in the program, and were treated and followed for several years.

During the study period, the eyes that converted to nAMD lost < 1 line of vision and a mean (SD) and a median (interquartile range) of 4.5 (11.5) and 4 (10.0–0.0) letters, respectively, at time of diagnosis of nAMD. From that point on, those eyes were treated consistently for a mean of 2.7 (2.0) years, with 25% of eyes for > 3.7 years, and with more than an annual mean of 6 anti-VEGF injections, an average rate that did not decline over the years, resulting in stable vision over the treatment years with no additional median vision loss. Over 80% of eyes maintained functional vision of 20/40 or better at the most final study follow-up, supporting the study hypothesis.

These findings are consistent with independent findings from early diagnostics and long-term outcomes studies previously reported. The HOME study^14^ and the real-world performance study^19^ both found a vision loss of < 1 line of vision from baseline to conversion to nAMD when using the home monitoring strategy. The Intelligent Research in Sight^26^ database and other studies^22,23^ reported a stable vision from conversion to 2 years in patients with a VA of 20/40 at initiation of anti-VEGF therapy. In this study, we were able to connect these 2 segments of iAMD and nAMD disease by collecting and reporting longitudinal information about the integrated implementation of a preferred monitoring and management scheme for these eyes, beginning with a prescription for FSH home monitoring, followed by early detection of conversion to nAMD, and ending with a consistent, long-term treatment with anti-VEGF therapy.

Over time, Comparison of AMD Treatments Trials and Intelligent Research in Sight^8,26^ grouped the VA as approximately parallel lines that never cross; that is, the vision at conversion best predicts the vision long term. We found that the home monitoring program resulted in 84% of eyes in the upper group of VAs of 20/40 or better with the best prognosis. In contrast, real-world Intelligent Research in Sight data showed that only 34% of eyes are presented with this opportunity under standard of care.^26^

The annual conversion rate in this study was found to be 2.72%, which is within the expected range for the iAMD population when compared with the reported risk levels in the AREDS study.^27,28^ The study found that participants tested with the device > 5 times per week consistently over long periods of time reaching 10 years. By doing so, they provided indirect evidence that, with the ongoing support from the NVMC and physicians, patients understood both the importance of monitoring and the ease of the device used to have it integrated into their daily routine. The Kaplan–Meier survival analysis resulted in a predicted mean monitoring duration of 4.5 years, which is a significant amount of time for the aging AMD population. Non-nAMD alerts were issued to the participants on average once every 4.6 years, reflecting a reasonable burden of unscheduled office visits to this population. Once diagnosed with nAMD and transition to the treatment phase occurred, the follow-up percentage observed in the study was high, with no reported office visit for only approximately 10% of patients in the last 6 to 12 months before the data lock.

A “number need to monitor” analysis, equivalent to number needed to treat, shows that, with a 2.7% annual conversion rate and 52% of detections first triggered by device alert, 71.4 participants had to be monitored for 1 year to yield a single detection triggered by a device alert. The FSH monitoring program is an integrated monitoring strategy with several elements that cannot be easily isolated. Monitoring with the device adds a safety net of suspected change alerts to bolster other existing means of prompting a detection of conversion to nAMD.

Several factors that cannot be isolated affect the distribution of detection between the modalities of device alerts, symptoms realization, and prescheduled office visits. The device allows for improved hyperacuity due to continuous testing with the system and constant awareness of the risks associated with iAMD that may enhance symptoms realization. In contrast, reliance on the technology by the participant that the system will alert about any change can potentially create a sense of denial that may work to suppress subjective symptom changes.

The 52% of device-triggered detections of conversions is consistent with the 51% reported from the HOME study.^28^ In addition to detections during routine office visits, it seems that, in those cases where the patient did not notice a change in vision, FSH detected a conversion at the same VA, resulting in the same number of injections compared with symptom realization. As such, FSH eliminated the low end of the distribution of change in VA from baseline to time of conversion. Half of the converted eyes benefited from FSH alerts, whereas the other half never hit the safety net.

The frequency of routine visits to the clinic is strongly related to the status of the fellow eye, as visits for nAMD typically include simultaneous eye examinations and imaging of the iAMD eye with OCT, which can lead to earlier detection. In this study, the nAMD status of the fellow eye was recorded and used to test the hypothesis that subjects with 1 eye already affected by nAMD are more likely to realize symptoms and/or have a higher frequency of in-office examinations and therefore would have more conversion events detected without an FSH device alert. We found that there was an increase in the proportion of nAMD events detection triggered by symptom realization or standard care visits from 44% with dry-AMD fellow eye to 53% with nAMD fellow eye, however. The difference was not statistically significant (*P* = 0.12). The remaining 47% of nAMD fellow eyes benefited from the FSH safety net, prompting early initiation of treatment and supporting overall visual prognosis. Remote monitoring of fellow eyes may prove particularly relevant in the future as patients transition to receiving longer acting treatments in their nAMD eye, leading to reduced number of office visits.

Ho et al^8^ analyzed several publications and integrated anatomic and functional findings, such as lesion growth and degradation of vision, and suggested that early detection may allow initiation of treatment 6 to 12 months earlier than current standard care practices. Studies^21–23,29^ have shown that, when eyes with nAMD are treated consistently over time, vision is maintained, whereas poor VA is one of the main causes for lack of follow-up. Previous health economy analysis of the FSH concluded that home telemonitoring of patients with AMD who are at risk for choroidal neovascularization was cost effective when compared with scheduled examinations alone.^30^ Furthermore, the economic burden of eye care is projected to increase significantly in coming years, with most of these costs attributable to the indirect costs of vision loss.^30–32^ ForseeHome supports the extension of the effective therapeutic window, with an earlier start and longer maintenance of treatment, and therefore can improve the overall visual outcomes and ultimately the return on the healthcare investment. The data about proportion of eyes that established a baseline before entry into the study indicated a higher rate than was previously reported in another study,^33^ with 82.9% versus 73.7%, respectively.

A limitation of this study was that prequalification information about the number of prescriptions filled by the patients was not collected. The VA data for eyes that did not convert were only available for 30% to 40% of the eyes, but this was still at least 900 eyes at any time point. Due to its retrospective nature, VA measurements methods were of standard clinical care with Snellen acuity charts and not with the strict study methodology used in clinical trials. The study did not collect information about the drugs used for treatment and the management scheme, for example, pro re nata or treat and extend.

Strengths of the study include the large sample size of all eyes that enrolled in 5 high-prescribing clinics, representing a practical, real-world approach to the selection of candidates for receiving a prescription, as well as the long duration of follow-up throughout the disease process. The 10-year anniversary of the AREDS2 study allowed for long-term replication of its original findings.^34^ The data lock for the ALOFT study also took place 10 years after the initiation of enrollment to the HOME study, allowing us to validate the long-term outcomes of the original findings from that AREDS2 ancillary study.

Patients who participated in the FSH monitoring program showed excellent VA at the time of conversion to nAMD, were treated consistently, and maintained good vision long term, emphasizing the importance of early detection and treatment in this potentially visually devastating disease.

## Figures and Tables

**Table 1.** Baseline Characteristics and Monitoring Duration by Study Site

	Study Center					
		
	*Elman Retina Group*	*Retina Associates of Kentucky*	*Retina Group of Washington*	*Virginia Eye Institute*	*Wagner Macula & Retina Center*	Total/Mean/SD

No. (%) of participants	614 (29)	201 (9)	638 (30)	477 (22)	193 (9)	2123
No. (%) of eyes	995 (30)	307 (9)	964 (29)	789 (24)	279 (8)	3334
Age (SD) at baseline (yrs)	73 (8)	74 (8)	75 (8)	76 (7)	75 (7)	74 (8)
No. (%) of females	347 (57)	138 (69)	402 (63)	304 (64)	111 (58)	1302 (61)
Mean (SD) monitoring years per participant	3.9 (2.9)	2.3 (1.6)	3.4 (2.4)	2.7 (1.8)	2.2 (1.7)	3.1 (2.4)

SD = standard deviation.

**Table 2.** VA Outcomes at Baseline, at Conversion, and at Recent Visit

	Eyes That Converted While Monitored	Eyes That Did Not Convert While Monitored	P Value^‡^	All Eyes

No. of eyes (N)	285	3049		3334
At baseline				
n (%) of eyes with VA data VA	279 (98)	1134 (37)		1413 (42)
Mean (SD): LogMAR	0.19 (0.16)	0.17 (0.17)		0.17 (0.17)
Mean: Snellen equation	20/31	20/30		20/30
Median (IQR): LogMAR	0.18 (0.1–0.3)	0.13 (0.02–0.26)	0.014	0.14 (0.04–0.3)
Median: Snellen equation	20/30	20/27		20/28
% with VA ≥20/40 (95% CI)*	86 (81–90)	87 (85–89)		87 (85–88)
At conversion to neovascular AMD				
n (%) of eyes with VA data VA	282 (99)	N/A		N/A
Mean (SD): LogMAR	0.28 (0.22)	N/A		N/A
Mean: Snellen equation	20/38	N/A		N/A
Median (IQR): LogMAR	0.29 (0.1–0.4)	N/A		N/A
Median: Snellen equation	20/39	N/A		N/A
% maintained VA ≥20/40 (95% CI)*	84 (78–88)	N/A		N/A
VA change from baseline				
Mean (SD): LogMAR	−0.09 (0.23)	N/A		N/A
Mean – Letters^†^	−4.5 (11.5)	N/A		N/A
Median (IQR): LogMAR	−0.08 (−0.2 to 0.0)	N/A		N/A
Median: letters	−4.0 (−10.0 to 0.0)	N/A		N/A
Time from baseline (yrs)				
Mean (SD)	2.3 (1.9)	N/A		N/A
Median (IQR)	1.8 (0.8–3.2)	N/A		N/A
At recent visit				
n (%) of eyes with VA data VA	283 (99)	901 (30)		1184 (36)
Mean (SD): LogMAR	0.32 (0.36)	0.23 (0.29)		0.25 (0.31)
Mean: Snellen equation	20/42	20/34		20/36
Median (IQR): LogMAR	0.2 (0.1–0.41)	0.18 (0.1–0.3)	<0.001	0.18 (0.1–0.3)
Median: Snellen equation	20/32	20/30		20/30
% maintained VA ≥20/40 (95% CI)	82 (76–86)	85 (82–87)		84 (82–86)
VA change from baseline				
Mean (SD): LogMAR	−0.14 (0.36)	−0.07 (0.31)		−0.09 (0.32)
Mean: letters	−7.0 (0.18)	−3.5 (15.5)		−4.5 (16.0)
Median (IQR): LogMAR	−0.08 (−0.22 to 0.06)	0.00 (−0.18 to 0.08)	0.004	0.00 (−0.18 to 0.08)
Median: letters	−4.0 (−11.0 to 3.0)	0.0 (−9.0 to 4.0)		0.0 (−9.0 to 4.0)
Time from baseline (yrs)				
Mean (SD)	5.0 (2.3)	4.3 (2.4)		4.4 (2.36)
Median (IQR)	4.5 (3.1–6.6)	4.2 (2.4–6.1)		4.3 (2.57–6.22)
VA change from conversion				
Mean (SD): LogMAR	−0.04 (0.35)	N/A		N/A
Mean: letters	−2.0 (17.5)	N/A		N/A
Median (IQR): LogMAR	0.00 (−0.1 to 0.1)	N/A		N/A
Median: letters	0.0 (−5.0 to 5.0)	N/A		N/A
Time from conversion (yrs)				
Mean (SD)	2.7 (2.0)	N/A		N/A
Median (IQR)	2.3 (1.0–3.7)	N/A		N/A

CI = confidence interval; IQR = interquartile range; LogMAR = logarithm of the minimum angle of resolution; N/A = not applicable; SD = standard deviation; VA = visual acuity.

* LogMAR cutoff for VA≥20/40 is 0.35.

† 0.02 LogMAR=1 ETDRS letter.

‡ Mann–Whitney test.

## Abbreviations and Acronyms:

ALOFT: Analysis of the Long-term Visual Outcomes of ForeseeHome Remote Telemonitoring
AMD: age-related macular degeneration
FSH: ForseeHome
HOME: Home Monitoring of the Eye
iAMD: intermediate AMD
nAMD: neovascular AMD
NVMC: Notal Vision Monitoring Center
SD: standard deviation
VA: visual acuity
